# Silencing of SNHG12 Enhanced the Effectiveness of MSCs in Alleviating Ischemia/Reperfusion Injuries via the PI3K/AKT/mTOR Signaling Pathway

**DOI:** 10.3389/fnins.2019.00645

**Published:** 2019-06-25

**Authors:** Yuanzhi Li, Shenquan Guo, Wenchao Liu, Tao Jin, Xifeng Li, Xuying He, Xin Zhang, Hengxian Su, Nan Zhang, Chuanzhi Duan

**Affiliations:** ^1^The National Key Clinical Specialty, The Engineering Technology Research Center of Education Ministry of China, Guangdong Provincial Key Laboratory on Brain Function Repair and Regeneration, Department of Neurosurgery, Zhujiang Hospital, Southern Medical University, Guangzhou, China; ^2^Department of Neurosurgery, Affiliated Hengyang Hospital, Southern Medical University (Hengyang Central Hospital), Hengyang, China

**Keywords:** ischemia/reperfusion injury, SNHG12, mesenchymal stem cell, apoptosis, autophagy

## Abstract

Previous studies have reported that the long non-coding RNA SNHG12 (lncRNA SNHG12) plays a critical role in regulating the function of mesenchymal stem cells (MSCs); however, the effect of lncRNA SNHG12 on MSCs in injured brain tissue has rarely been reported. We studied the effect and mechanism of lncRNA SNHG12-modified mesenchymal stem cells (MSCs) in treating brain injuries caused by ischemia/reperfusion (I/R). I/R treated rat brain microvascular endothelial cells (BMECs) were co-cultured with MSCs or I/R pretreated MSCs. Next, BMEC proliferation was detected by using CCK-8 and EdU assays, and cell apoptosis was determined by using flow cytometry and the Hoechst staining method. Autophagy of BMECs was determined using immunofluorescence and expression of associated pathway proteins were measured by western blotting. Moreover, BMEC proliferation, apoptosis, and autophagy were also determined after the BMECs had been co-cultured with shSNHG12-MSCs. In addition, a rat model of middle cerebral artery occlusion (MCAO) was used to further confirm the findings obtained with cells. I/R treatment significantly decreased the proliferation of BMECs, but increased their levels of SNHG12 expression, apoptosis, and autophagy. However, co-culturing of BMECs with MSCs markedly alleviated the reduction in BMEC proliferation and the increases in BMEC apoptosis and autophagy, as well as the phosphorylation of PI3K, AKT, and mTOR proteins in BMECs that had been induced by I/R. Furthermore, shSNHG12 remarkably enhanced the effects of MSCs. In addition, an injection MSCs reduced the infarct areas and rates of cell apoptosis in MACO rats, and reduced the phosphorylation of PI3K, AKT, and mTOR proteins. Moreover, shSNHG12 enhanced the ameliorative effect of MSCs in treating brain injuries in the MACO rats. In conclusion, silencing of SNHG12 enhanced the effects of MSCs in reducing apoptosis and autophagy of BMECs by activating the PI3K/AKT/mTOR signaling pathway.

## Introduction

Ischemic stroke, which is caused by an interruption of blood supply, is a leading cause of mortality and morbidity worldwide (Farrell-Dillon et al., 2017). Although the incidence of stroke in the United States has declined with the development of new treatment agents and intravascular techniques, the annual worldwide incidence and prevalence of stroke has continued to increase. Cerebral tissue damage that occurs after ischemia significantly threatens the survival and quality of life of stroke patients (Liu et al., 2015; Siket, 2016). Multiple mechanisms, such as inflammation, apoptosis, oxidative damage, and excitatory neurotransmitter disorder, are involved in cerebral ischemia/reperfusion (I/R) injuries (Rodrigo et al., 2013; Chen et al., 2014; Sharma et al., 2018). Numerous treatment agents, including UPE1-400 (Connell et al., 2016), Pannexin-1 (Sharma et al., 2018), and Ginsenoside-Rg1 (Xie et al., 2015), have been evaluated for their ability to reduce the severity of I/R injuries and improve stroke outcomes in animal models. However, the exact outcomes produced by these agents remain unclear, and require further confirmation in the clinic.

Mesenchymal stem cells (MSCs) exhibit a fibroblast-like morphology, and are characterized by their expression of CD105, CD90, and CD73 antigens under standard conditions. MSCs are capable of differentiation into osteoblasts, adipocytes, and chondrocytes when exposed to different stimuli (Von Bahr et al., 2012). Many studies have shown that MSCs not only play a critical role in modulating inflammatory responses, but also secrete cytokines, as well as microvesicles that help transport materials between cells (Selmani et al., 2010; Bruno et al., 2012). Due to these properties, MSCs have been used to treat I/R injuries in the brain, liver, kidneys, and heart (Rowart et al., 2015). MSCs have been shown to not only migrate and assume neural phenotypes in damaged tissues, but also to reduce neuro-degradation by inhibiting apoptosis and releasing neurotrophic factors and cytokines in cerebral tissues damaged by an I/R injury (Li et al., 2000; Bliss et al., 2010). However, the therapeutic effect of MSCs in treating cerebral I/R injuries remains unclear. For example, Tsai et al. reported that bone marrow-derived MSCs had no effect on the morphology and cell surface markers of cerebral stroke tissues (Tsai et al., 2014). Thus, further investigations are required to elucidate the effects of MSCs in treating cerebral I/R injuries.

Several complicated mechanisms are responsible for the ability of MSCs to improve a cerebral I/R injury; these mechanisms include peripheral immuno-inflammation modulation (Cheng et al., 2015), proteomic complementation management (Lai et al., 2013), and apoptosis regulation (Li et al., 2012). Hypoxia treatment has been documented to improve the therapeutic efficacy of MSCs (Rosová et al., 2008). Long non-coding RNAs (lncRNAs) are a class of non-coding mRNA transcripts with a length of >200 nt. Previous studies have shown that the expression of lncRNA SNHG12 becomes significantly upregulated after an ischemic stroke (Ren and Yang, 2018) or a focal cerebral ischemic event (Zhang et al., 2016). Recently, another study showed that the levels of lncRNA SNHG12 expression in microvascular endothelial cells in mouse brain tissues were significantly increased after I/R or OGD/R (Zhao et al., 2018). However, it has rarely been investigated how lncRNA SNHG12 might facilitate the ability of MSCs to treat I/R injuries. In the current study, we investigated the mechanism by which MSCs treat I/R injuries at the molecular level, and utilized modified MSCs to optimize the therapeutic effect of MSCs. In addition, we confirmed the effect of the modified MSCs in an animal model. We hope our results provide a new MSC-based therapeutic method for improving the outcomes of patients with a cerebral I/R injury.

## Materials and Methods

### Culturing of MSCs and BMECs

Mesenchymal stem cells were purchased from Procell (CP-R108, Wuhan, China) and maintained in a complete cell culture medium (CM-R108). BMECs were also purchased from Procell (CP-R131) and cultured in a different complete medium (CM-R131). All cells were maintained at 37°C in a humidified environment with 5% CO_2_. ShSNHG12 and its corresponding negative control plasmid were purchased from GenePharma (Shanghai, China). Ethics approval and consent to participate: all experimental procedures were approved by the Southern Medical University Ethics Committee and were performed in accordance with the guidelines of the National Institutes of Health on the Care and Use of Animals.

### Transfection of MSCs

Mesenchymal stem cells were seeded into a 6-well plates at a density of 1.0 × 10^5^ cells per well. ShSNHG12 or its corresponding negative control plasmid was transfected into the MSCs by using Lipofectamine 2000 (Invitrogen, Carlsbad, CA, United States) according to manufacturer’s protocol. After 24 h of transfection, the MSCs were harvested and co-cultured with BMECs for the following experiments.

### I/R Treatment and Co-culture

For I/R treatment, cells were seeded into culture plates and exposed to I/R conditions as previously described (Yuan et al., 2017). Briefly, the BMECs were seeded into 6-well plates at a density of 5 × 10^5^ cells per well, and then maintained in DMEM supplemented with 10% FBS. The cell medium in each well was changed to serum-free medium at 24 h before treatment. Next, the ischemia/hypoxia medium was added to the cells, and plates were placed into double layered, sealed, self-styled bags (MGC AnaeroPack Series, Mitsubishi Gas Chemical Company, Tokyo, Japan), which were transferred into an anaerobic indicator filled with low-oxygen gas (5% CO_2_). After 2 h of incubation, oxygen was added to the bags, and medium for subsequent culturing was changed to DMEM with 10% FBS. For I/R treatment of MSCs, cells were maintained in 12-well plates that contained DMEM supplemented with 10% FBS prior to being co-cultured with BMECs in a double-chamber co-cultured system, which consisted of an upper chamber and a lower chamber separated by a 0.4 μm pore-size membrane.

### Induction of an I/R BMECs Model and Co-culture

The BMECs were divided into the following four groups: (1) BMEC group (normal BMECs); (2) BMEC+I/R group, (I/R treated BMECs); (3) BMEC+I/R+MSC group (I/R-treated BMECs co-cultured with MSCs); (4) BMEC+I/R+MSC-I/R group (I/R treated BMECs co-cultured with I/R treated MSCs).

To detect the role played by SNHG12 in I/R injuries, the BMECs were divided into the following five groups: (1) BMEC group (normal BMECs); (2) BMEC+I/R group, (I/R treated BMECs); (3) BMEC+I/R+MSC group (I/R treated BMECs co-cultured with MSCs); (4) BMEC+I/R+MSC-NC group (I/R treated BMECs co-cultured with MSCs that had been transfected with a negative control siRNA); (5) BMEC+I/R+MSC-siRNA group (I/R treated BMECs co-cultured with MSCs transfected with si-SNHG12).

### Immunofluorescence

Brain microvascular endothelial cells that had undergone the different treatments for 48 h were seeded into 6-well plates, washed with phosphate buffer solution (PBS), and then fixed with 4% paraformaldehyde (PFA) at 4°C overnight. Next, the cells were washed twice with PBS (3 min per wash), blocked with 10% goat serum for 15 min, and incubated with LC3B antibodies (1:200, Abcam, Cambridge, MA, United States) or P62 antibodies (1:50, Abcam, Cambridge, MA, United States) at 4°C for 1 h. After being washed three times with PBS (3 min per wash), the cells were incubated with Alexa Fluor 488 (for P62) or Alex Fluor 594 (for LC3B) conjugated secondary antibodies (1:1000, Abcam, Cambridge, MA, United States) at room temperature for 1 h. Next, the secondary antibodies were removed, and the cells were washed three times with PBS (5 min per wash). Finally, the cells were mounted with anti-fluorescence quench sealing liquid, and analyzed under an inverted florescence microscope (Bx51. Olympus Corporation, Shinjuku, Japan).

### CCK-8 and EdU Assays

Cell viability was determined by using CCK-8 and EdU assays. BMECs were seeded into 96-well plates at a density of 1.0 × 10^3^ cells per well and subsequently cultured in complete DMEM medium, followed by I/R treatment after adherence. After I/R treatment, the cells were maintained for periods of 0 h, 24 h, and 48 h, respectively. When conducting the CCK-8 assay, the cell supernatants were discarded, and 1 mL of DMEM medium containing 10 μL of CCK-8 solution (Dojindo, Japan) was added to each well and incubated at 37°C for 1 h. Next, the plates were shaken for 5 min at room temperature; after which, the optical density (OD) of each well was determined at 450 nm. When conducting the EdU assay, the original cell culture medium was replaced with fresh medium containing 0.1% EdU buffer A, and used to culture the cells for 2 h. After culturing, the cells were harvested and centrifuged at 350g for 5 min, washed with PBS, centrifuged again, and then fixed with 4% PFA at room temperature for 15∼30 min. Next, the cells were centrifuged at 600 *g* for 10 min, and fixation was terminated by addition of 2∼3 mL of 2 mg/mL glycine (Sigma), followed by washing in PBS. Subsequently, the cells were incubated in 0.5% Triton X-100 (in PBS) solution at room temperature for 10 min, and washed one time with PBS. The washed cells were then resuspended in 1× Apollo staining solution, incubated at room temperature for 10 min, washed three times with 0.5% Triton X-100 (in PBS) solution, and resuspended in PBS. All procedures were performed according to instructions provided with a Cell-Light^TM^ EdU Apollo^®^ 488 In Vitro Flow Cytometry Kit (RiboBio, Guangzhou, China). After completing these procedures, cell viability was determined by flow cytometry (BD, FACSCalibur, San Jose, CA, United States).

### Construction of an I/R Model and MSC Transplantation

Thirty-six Sprague-Dawley (SD) rats (aged 7∼8 weeks) were purchased from the Guangdong Medical Laboratory Animal Center (Foshan, China) and used to assess the therapeutic effects of MSCs on I/R injuries in a rat model of middle cerebral artery occlusion (MCAO). After adaptation, the rats were randomly assigned to the following six groups (*n* = 6 rats per group): (1) control group (without treatment), (2) sham group, (3) MCAO model group, (4) MCAO+MSC, (5) MCAO+MSC-shRNA-NC, and (6) MCAO+MSC-shRNA-SNHG12. For the MCAO model, rats were anesthetized by an intraperitoneal injection of 10% chloral hydrate (3.5 mL/kg) into a region sterilized with iodine. Next, a ventral midline incision was made to expose the right common carotid artery (CCA), internal carotid artery (ICA), and external carotid artery (ECA); after which, a 4-0-monofilament nylon suture with a 0.26 mm diameter was prepared and inserted into the right CCA lumen and gently advanced into the ICA up to a point ∼18 mm distal to the bifurcation of the carotid artery. Reperfusion was achieved by slowly retracting the suture after 90 min of occlusion. Subsequently, the incision was sutured, and the animal was allowed to recover. For the sham group, the CCA, ICA, and ECA were exposed, and the incision was subsequently closed without insertion of a nylon suture. Rats in the MSC treatment groups were stereotactically injected with 2 × 10^6^ MSCs (MSC-shRNA-NC or MSC-shRNA-SNHG12) in a volume of 200 μL at 15 min prior to MCAO model construction, as previously described (Zhang et al., 2018). Two weeks later, the rats were sacrificed and their brain tissues were harvested for use in subsequent investigations.

### Hematoxylin-Eosin (H&E) Staining

To observe changes that occurred in brain tissue morphology, paraffin embedded sections of rat brain tissue were stained with H&E solution. Briefly, the rats in each group were sacrificed and their brains were removed. The brains were then dehydrated by exposure to decreasing concentrations of ethanol, embedded in paraffin wax, and cut into 5 μm thick sections. The sections were deparaffinized, and then rehydrated in decreasing concentrations of ethanol; after which, they were stained with hematoxylin (Servicebio, Wuhan, China) and eosin (Servicebio, Wuhan, China) according to the manufacturer’s protocols.

### Triphenyl Tetrazolium Chloride (TTC) Staining

After rats sacrificed, the brains were rapidly harvested and cut into 1-mm thick slices. Then, slices immersed into 2% TTC solution for 30 min at 37°C and were fixed with 4% paraformaldehyde for 30 min at room temperature. The brain slices were then photographed and analyzed by using Photoshop software as previously reported (Yang et al., 1998). Tissue slice without TTC staining was considered as core, and pink brain tissue section was penumbra positioned between core (Bederson et al., 1986). Specifically, red color represents the viable tissue and pale color represents the infarction tissue (Gill et al., 1995). Brain infarct size (%) were calculated by white infarct area/whole slice area × 100%.

### TUNEL Assay

TUNEL assays were performed with an In Situ Cell Death Detection Kit according to the manufacturer’s instructions. Briefly, the cells were fixed with 4% paraformaldehyde in 0.1 M phosphate buffer (pH 7.4). Next, the cells were washed with PBS, and then permeabilized with 0.2% Triton-X 100 in methanol for 2 min at 4°C; after which, they were incubated with TUNEL assay solution at 37°C for 60 min. Finally, the cells were washed with PBS and mounted with fluorescent mounting medium. The number of TUNEL-positive cells was obtained by counting the cells in six randomly selected microscopic fields as viewed with a 20× objective lens. The percentage of TUNEL-positive cells in each field was calculated and an average percentage for all 6 fields was obtained.

### Apoptosis Assay

The cell cycle phases and apoptosis rates of BMECs were determined by using flow cytometry. Briefly, BMECs were seeded in a 6-well plates, allowed to adhere, and then treated with ischemia for 48 h, followed by reperfusion for 12 h. Next, the BMECs were co-cultured with MSCs. After co-culture, the cells were harvested, washed twice with cold PBS, and resuspended in 200 μL of Annexin V/PI (MultiSciences, Hangzhou, China) for 15 min in the dark at room temperature. The cells were then analyzed by flow cytometry (BD, FACSCalibur, San Jose, CA, United States).

### Hoechst Assay

The Hoechst staining assay was used to detect the apoptosis of cells in different groups. Briefly, BMECs were seeded in a 6-well plates, allowed to adhere, and then treated with ischemia for 48 h, followed by reperfusion for 12 h. Next, the BMECs were co-cultured with MSCs. Following co-culture, the cells were fixed with 4% PFA at room temperature for 30 min, washed twice with PBS (3 min per wash), and then incubated with 20 μM Hoechst 33258 (Beyotime, Shanghai, China) at room temperature for 5 min. After incubation, the staining solution was discarded, the cells were washed twice with PBS (3 min per wash), and subsequently analyzed with an inverted florescence microscope (MOTIC AE2000, Ted Pella, Inc., Redding, CA, United States).

### Quantitative Real-Time PCR

TRIzol reagent (Takara, Dalian, China) was used to isolate the total RNA from cells, and the quality of the isolated RNA was verified using a Q6000 UV spectrophotometer (Quawell Technology, San Jose, CA, United States). Next, 2 μg of total RNA was reverse transcribed to cDNA by using a Bestar qPCR RT Kit (DBI, Shanghai, China) according to manufacturer’s instructions. Quantitative PCR (qPCR) was performed on an ABI 7500 Real-Time PCR System (Applied Biosystems, Foster City, CA, United States) and using a SYBR Green Real-time PCR assay kit (DBI, Shanghai, China). The conditions used for RT-PCR were as follows: 95°C for 2 min, followed by 40 cycles of 94°C for 20 s, 54°C for 20 s, and 72°C for 20 s. The primers used for RT-PCR were as follows: SNHG12-forward, 5′-GCCATGGTTCCCTTACTGATAC-3′ and SNHG12-reverse, 5′-CAGGTCCTCCATGATGTGTTCTA-3′; GAPDH-forward, 5′-TGTTCGTCATGGGTGTGAAC-3′; and GAPDH-reverse, 5′-ATGGCATGGACTGTGGTCAT-3′. Gene expression levels were quantitated using the 2^–ΔΔCt^ method (Livak and Schmittgen, 2012). All samples were analyzed in triplicate, and the mean value was calculated.

### Western Blotting

After removing the incubation medium, the treated cells were harvested and transferred into RIPA lysis buffer (Beyotime, Shanghai, China) that contained a proteinase inhibitor cocktail (0.01%, Sigma, St. Louis, MO, United States). For brain tissue, 0.05 mg of tissue was lysed in RIPA lysis buffer. Next, the lysates were centrifuged at 12,000 *g* for 10 min at 4°C; after which, the supernatant fractions were harvested and their protein concentrations were determined by using the BCA method (Prod, CA, United States). Next, a 20 μg sample of total protein was separated by SDS-PAGE, and the protein bands were electrophoretically transferred onto PVDF membranes, which were subsequently blocked with 5% bovine serum albumin (BSA, Sangon Biotech, Shanghai, China) at room temperature for 1 h. The membranes were then incubated with specific primary antibodies against SNHG12, P62, LC3B, caspase 3, GAPDH, p-AKT, p-PI3K, and p-mTOR, respectively (Abcam, Cambridge, MA, United States) at 4°C overnight. After incubation, the membranes were washed three times with TBST, and then incubated with Goat anti-rabbit/mouse secondary antibodies (Boster, Wuhan, China) at room temperature for 40 min. Finally, the membranes were washed and then visualized using an ECL-detection system (PerkinElmer, Boston, MA, United States).

### Statistical Analysis

All statistical analyses were performed using GraphPad Prism (7.0) software. Comparisons among multiple groups were assessed using ANOVA followed by Turkey’s multiple comparisons test. Statistical results are presented as the mean ± standard deviation (SD). For all comparisons, a *P*-value <0.05 was considered statistically significant.

## Results

### MSCs Improved the Proliferation of BMECs After I/R

**FIGURE 1:**
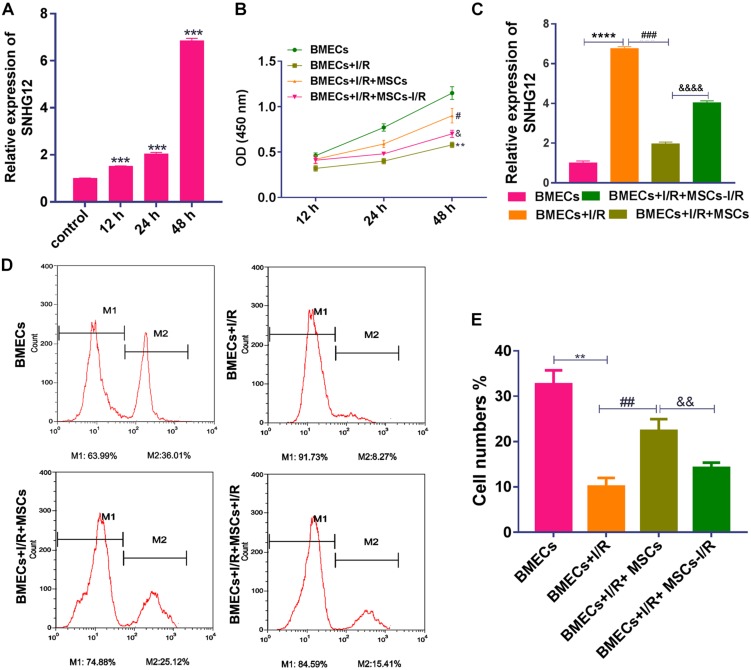
Co-culturing with MSCs promoted the proliferation of BMECs after I/R treatment. **(A)** Relative expression of SNHG12 in BMECs as determined by qRT-PCR. **(B)** Proliferation of BMECs as determined by the CCK-8 assay. **(C)** Relative expression of SNHG12 in BMECs co-cultured with MSCs as determined by qRT-PCR. **(D)** Cell proliferation as determined by the EdU assay. **(E)** Cell proliferation as determined by the EdU assay. The percentage of the M2 peak was analyzed as the percentage of EdU-positive cells. MSCs, mesenchymal stem cells; BMECs, brain microvascular endothelial cells; I/R, ischemia/reperfusion. Comparisons among multiple groups were assessed using ANOVA followed by Turkey’s multiple comparisons test. Compared with the control group, ^∗∗^*P* < 0.01, ^∗∗∗^*P* < 0.001, and ^****^P < 0.0001; compared with the I/R group, ^#^*P* < 0.05, ^##^*P* < 0.01, and ^###^*P* < 0.001; compared with the I/R+MSCs group, ^&^*P* < 0.05, ^&⁣&^*P* < 0.01, and ^&⁣&⁣&⁣&^*P* < 0.0001.

After treatment with I/R, SNHG12 expression was significantly increased (*P* < 0.001; Figure 1A). To further confirm our finding, the expression of SNHG12 was silenced in BMEC (Supplementary Figure S1A) and the cell behaviors of BMECs were determined. The results showed that silencing SNHG12 could significantly promote the apoptosis, but inhibit the proliferation ability of BMECs after I/R treatment (Supplementary Figures S1B,C), indicating that SNHG12 might play critical role in responding to I/R injury. To explore the effects of MSCs in treating with I/R injury, the CCK-8 assay was used to determine BMEC proliferation after I/R treatment and/or after the BMECs had been co-cultured with MSCs. The results showed that I/R treatment significantly decreased the proliferation of BMECs, while co-culture with MSCs markedly alleviated that reduction (all *P* < 0.001; Figure 1B). In addition, MSCs also decreased the expression of SNHG12 in BMECs treated with I/R, and MSCs pre-treated with I/R alleviated that reduction (all *P* < 0.001; Figure 1C). EdU assays revealed that co-cultured MSCs without I/R treatment remarkably attenuated the reduction in BMEC proliferation induced by I/R, and MSCs pre-treated with I/R impaired that effect of the MSCs (all *P* < 0.01; Figures 1D,E).

### Co-cultured MSCs Inhibited I/R-Induced Apoptosis and Autophagy in BMECs

**FIGURE 2:**
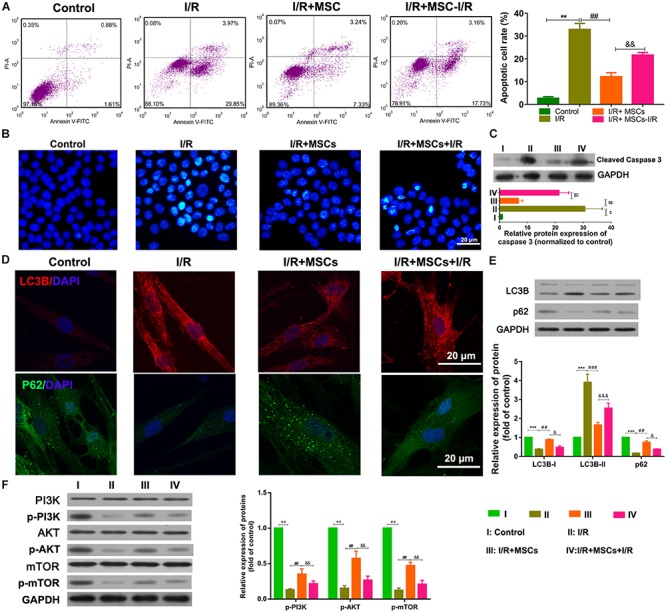
Mesenchymal stem cells reduced BMEC apoptosis and autophagy via the PI3K/AKT/mTOR pathway. **(A)** Apoptosis of BMECs as determined by flow cytometry. **(B)** Apoptosis of BMECs as determined by Hoechst staining. **(C)** Expression of caspase 3 as determined by western blotting. **(D)** Expression of LC3B (red) and P62 (green) in BMECs as determined by immunofluorescence. **(E)** Expression of LC3B and p62 as determined by western blotting. **(F)** Expression and phosphorylation of PI3K, AKT, and mTOR in BMECs as determined by western blotting. MSCs, mesenchymal stem cells; BMECs, brain microvascular endothelial cells; I/R, ischemia/reperfusion. Comparisons among multiple groups were assessed using ANOVA followed by Turkey’s multiple comparisons test. Compared with the control group, ^∗∗^*P* < 0.01 and ^∗∗∗^*P* < 0.001; compared with the I/R group, ^##^*P* < 0.01 and ^###^*P* < 0.001; compared with the I/R+MSC group, ^&^*P* < 0.05, ^&⁣&^*P* < 0.01, ^&⁣&⁣&^*P* < 0.001.

The apoptosis and autophagy of BMECs were also examined after co-culture with MSCs. I/R treatment significantly increased the apoptosis of BMECs, while co-culture with MSCs significantly suppressed that increase in apoptosis (all *P* < 0.01, Figure 2A). The same result was also shown by the Hoechst assay (Figure 2B). Western blot studies revealed that co-culture with MSCs also inhibited the upregulated expression of caspase 3 in BMECs induced by I/R (all *P* < 0.01; Figure 2C). However, MSCs treated with I/R impaired the ameliorating effect of MSCs on BMECs after I/R. Immunofluorescence analyses showed that I/R treatment significantly increased LC3B expression but decreased p62 expression in BMECs, while co-culture with MSCs markedly alleviated those changes in expression (Figure 2D). Western blot assay also revealed that I/R treatment significantly accumulated LC3B expression but reduced p62 expression in BMECs, while co-culture with MSCs obviously alleviated those changes in expression of LC3B and p62 (all *P* < 0.01; Figure 2E). Western blot studies revealed that I/R treatment significantly decreased the phosphorylation levels of PI3K, AKT, and mTOR proteins, while MSC treatment significantly suppressed those reductions in phosphorylation (all *P* < 0.01, Figure 2F).

### Silencing of SHNG12 in MSCs Increased the Proliferation of Co-cultured BMECs

**FIGURE 3:**
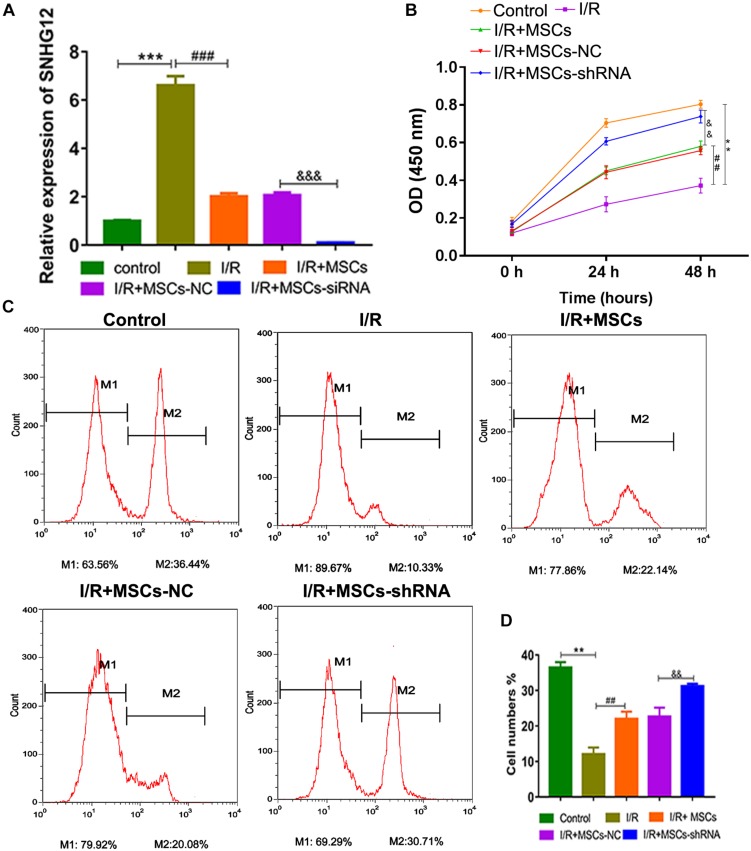
Silencing of SNHG12 enhanced the effect of MSCs in promoting the proliferation of BMECs after I/R. **(A)** Relative expression of SNHG12 in BMECs after being co-cultured with different MSCs, as determined by qRT-PCR. **(B)** Proliferation of BMECs after being cultured with different MSCs, as detected by the CCK-8 assay. **(C)** Cell proliferation as determined by the EdU assay. **(D)** Cell proliferation as determined by the EdU assay. MSCs, mesenchymal stem cells; BMECs, brain microvascular endothelial cells; I/R, ischemia/reperfusion; NC, negative control. Comparisons among multiple groups were assessed using ANOVA followed by Turkey’s multiple comparisons test. Compared with the control group, ^∗∗^*P* < 0.01 and ^∗∗∗^*P* < 0.001; compared with the I/R group, ^##^*P* < 0.01 and ^###^*P* < 0.001; compared with the I/R+MSC-NC group, ^&⁣&^*P* < 0.01 and ^&⁣&⁣&^*P* < 0.001.

For further investigation, SNHG12 was silenced in MSCs and co-cultured with BMECs, followed by SNHG12 expression detection. RT-qPCR results showed that I/R treatment significantly increased SNHG12 expression (*P* < 0.001), whereas the co-culturing of MSCs with BMECs significantly inhibited this that increase in SNHG12 expression (*P* < 0.001). Furthermore, shSHNG12 in MSCs could enhance that inhibitory effect on SNHG12 expression (*P* < 0.001, Figure 3A). CCK-8 assays revealed that MSCs significantly increased the proliferation of BMECs after I/R treatment, and silencing of SHNG12 in MSCs significantly enhanced that promotive effect on BMEC proliferation (all *P* < 0.01, Figure 3B). In addition, EdU assays also showed that MSCs ameliorated the reduction in BMEC proliferation induced by I/R, and suppression of SHNG12 expression in MSCs further the alleviated the inhibition of BMEC proliferation induced by I/R (all *P* < 0.01, Figures 3C,D).

### Silencing of SHNG12 in MSCs Decreased the Apoptosis and Autophagy of Co-cultured BMECs

**FIGURE 4:**
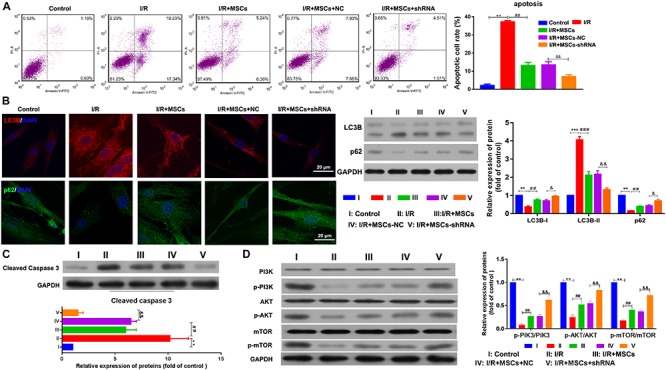
Silencing of SNHG12 enhanced the effect of MSCs in reducing apoptosis and autophagy of BMECs after I/R. **(A)** Apoptosis of BMECs after being co-cultured with different MSCs, as determined by flow cytometry. **(B)** Expression of LC3B (red) and P62 (green) in BMECs after being co-cultured with different MSCs, as determined by immunofluorescence at 400× and western blotting. **(C)** Expression and quantitative expression of caspase 3 as determined by western blotting. **(D)** Phosphorylation and quantitative phosphorylation of PI3K, AKT, and mTOR in BMECs, as determined by western blotting. MSCs, mesenchymal stem cells; BMECs, brain microvascular endothelial cells; I/R, ischemia/reperfusion. Comparisons among multiple groups were assessed using ANOVA followed by Turkey’s multiple comparisons test. Compared with the control group, ^∗∗^*P* < 0.01 and ^∗∗∗^*P* < 0.001; compared with the I/R group, ^##^*P* < 0.01 and ^###^*P* < 0.001; compared with the I/R+MSC-NC group, ^&^*P* < 0.05 and ^&⁣&^*P* < 0.01.

The apoptosis of MSCs that had been co-cultured with BMECs was also determined by flow cytometry. The results showed that co-culturing with MSCs significantly decreased the apoptosis of BMECs induced by I/R, and suppression of SHNG12 expression in MSCs significantly enhanced the reductions in BMEC apoptosis (all *P* < 0.01. Figure 4A). Both immunofluorescence assays and western blot showed that LC3B expression was significantly elevated in BMECs after I/R treatment, while co-culture with MSCs significantly decreased that elevation in LC3B expression (all *P* < 0.001, Figure 4B). Moreover, silencing of SNHG12 significantly enhanced the inhibitory effect of MSCs in reducing LC3B expression in co-cultured BMECs (*P* < 0.01; Figure 4B). In addition, co-culturing with MSCs also alleviated the reduction in p62 expression in BMECs that had been induced by I/R, and silencing of SNHG12 enhanced that alleviating effect of MSCs on BMECs (all *P* < 0.01; Figure 4B). Western blot studies revealed that I/R treatment significantly increased caspase 3 expression in BMECs, while co-culture with MSCs inhibited caspase 3 expression in BMECs, and downregulation of SNHG12 in MSCs significantly enhanced that inhibitory effect on caspase 3 expression in BMECs (all *P* < 0.01, Figure 4C). A pathway analysis revealed that I/R treatment significantly decreased the phosphorylation levels of PI3K, AKT, and mTOR proteins in BMECs, but co-culture with MSCs significantly increased those phosphorylation levels (all *P* < 0.01; Figure 4D). Silencing of SNHG12 significantly enhanced the promotive effect of MSCs on the phosphorylation of PI3K, AKT, and mTOR in BMECs (all *P* < 0.01; Figure 4D).

### Silencing of SNHG12 in MSCs Ameliorated Brain Injuries in MCAO Rats

**FIGURE 5:**
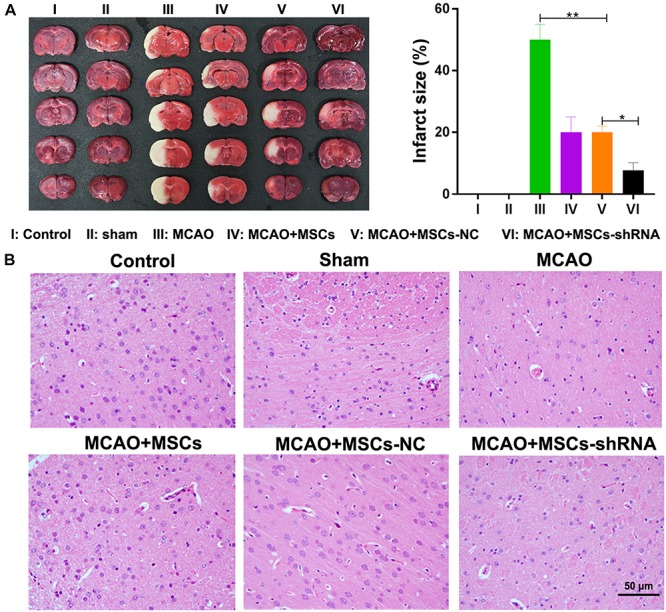
Silencing of SNHG12 enhanced the effect of MSCs in reducing the infarct area of MACO rat brain tissue. **(A)** Infarct areas in MACO rats as determined by the TTC method. **(B)** H&E staining of brain tissues from the MACO rats. Rats in the control group are treated without any treatment, in the sham group are exposed the right CCA, ICA, and ECA followed by sutured, in the MCAO group were artery occlusion and reperfusion to construct the I/R injury mode, and in rats in the MSC treatment groups were stereotactically injected with MSCs (MSC-shRNA-NC or MSC-shRNA-SNHG12) prior to MCAO model construction. CCA, common carotid artery; ICA, internal carotid artery; ECA, external carotid artery; MSCs, mesenchymal stem cells; BMECs, brain microvascular endothelial cells; I/R, ischemia/reperfusion; MCAO, middle cerebral artery occlusion; NC, negative control. Comparisons among multiple groups were assessed using ANOVA followed by Turkey’s multiple comparisons test. ^*^P < 0.05, ^∗∗^*P* < 0.01.

After considering our *in vitro* findings, we sought to confirm them *in vivo* using an MSC-treated MCAO rat model. Rats were injected with MSCs for 2 weeks; after which, their brains were harvested and investigated for morphological characteristics. TTC staining showed that I/R treatment induced obvious infarcts in the brains of the rats (*P* < 0.01); however, MSC treatment significantly decreased the areas of the brain infarcts, and silencing of SNHG12 significantly enhanced the ameliorative effect of MSCs in reducing the infarct areas (all *P* < 0.05, Figure 5A). Similar results were obtained by H&E staining (Figure 5B).

### Silencing of SNHG12 in MSCs Decreased Apoptosis and Autophagy in MACO Rat Brain Tissues

**FIGURE 6:**
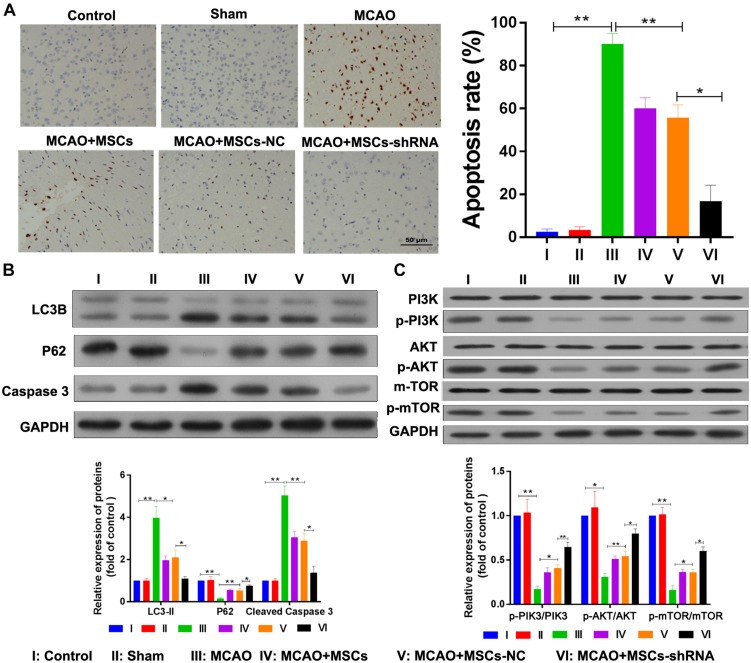
Silencing of SNHG12 enhanced the effect of MSCs in reducing apoptosis and autophagy in MACO rat brain tissue. **(A)** Apoptosis in MACO rat brain tissues as determined by the TUNEL method and magnified at 200×. **(B)** Expression of LC3B, P62, and caspase 3 in rat brain tissues as determined by western blotting. **(C)** Phosphorylation of PI3K, AKT, and mTOR in rat brain tissues. CCA, common carotid artery; ICA, internal carotid artery, ECA, external carotid artery; MSCs, mesenchymal stem cells; BMECs, brain microvascular endothelial cells; I/R, ischemia/reperfusion; MCAO, middle cerebral artery occlusion; NC, negative control. Comparisons among multiple groups were assessed using ANOVA followed by Turkey’s multiple comparisons test. ^*^P < 0.05 and ^∗∗^*P* < 0.01.

The apoptosis and autophagy of rat brain tissue were also determined after MSC treatment. TUNEL assays indicated that significant levels of apoptosis were occurring in the brains of rats in the MCAO model (*P* < 0.01); however, MSC injections inhibited that apoptosis (*P* < 0.01), and downregulation of SNHG12 remarkably enhanced the effect of MSCs in reducing apoptosis in the MCAO rat brains (*P* < 0.05, Figure 6A). Western blot studies showed that p62 expression was significantly decreased in the MCAO model group (*P* < 0.01), while LC3B and caspase 3 expression were significantly increased in the MCAO model group when compared with the sham group (all *P* < 0.01, Figure 6B). Moreover, MSC injections significantly elevated the levels of p62 expression but reduced the levels of LC3B and caspase 3 expression in the MCAO model group (all *P* < 0.05; Figure 6B). Furthermore, silencing of SNHG12 significantly enhanced the effect of MSCs in modulating the levels of p62, LC3B, and caspase 3 expression in the MCAO model group, indicating that shSNHG12 MSCs could significantly ameliorate autophagy in brain tissues induced by I/R (all *P* < 0.05, Figure 6B). A pathway analysis revealed that the phosphorylation levels of PI3K, AKT, and mTOR proteins were significantly downregulated in the MACO group when compared with those levels in the sham group (all *P* < 0.05, Figure 6C). However, MSC treatment significantly upregulated the phosphorylation levels of PI3K, AKT, and mTOR proteins in the MACO model rats, and shSNHG12 markedly enhanced the effect of MSCs in elevating the phosphorylation levels of PI3K, AKT, and mTOR proteins in those rats (all *P* < 0.01, Figure 6C). These findings suggest that shSNHG12 in MSCs might work via the PI3K/AKT/mTOR signaling pathway to alleviate the autophagy and apoptosis that occurs in rat brains induced by I/R.

## Discussion

Numerous studies have shown that MSCs play critical anti-inflammatory and neuroprotective roles in cerebral I/R injuries (Ma et al., 2013; Xin et al., 2013; Liu et al., 2014). However, their therapeutic effects are not fully understood, and findings made in *in vitro* studies and animal models are yet to be confirmed in the clinic. In the current study, SNHG12 levels in BMECs were significantly increased after I/R treatment. MSCs markedly reduced the apoptosis and autophagy of BMECs induced by I/R, and silencing of SNHG12 significantly enhanced the effect of MSCs. MSCs also demonstrated a significant ameliorative effect *in vivo*, as they reduced the infarct areas and levels of apoptosis in a MACO rat model. Furthermore, shSNHG12-MSCs demonstrated an even greater ameliorative effect than MSCs.

Small nucleolar RNA host gene 12 (SNHG12) is one of many long non-coding RNAs involved in the progression of several types of cancer. Wang O. et al., 2017 demonstrated that C-MYC upregulates SNHG12 expression to promote cell proliferation and reduce apoptosis in triple-negative breast cancer. LncRNA SNHG12 also promotes the growth and inhibits the apoptosis of colorectal cancer cells and papillary thyroid carcinoma cells (Wang J.Z. et al., 2017; Ding et al., 2018). Yin et al. (2019) and Li et al. (2017) reported that SNHG12 can interact with miR-199a to attenuate a cerebral I/R injury via the AMPK signaling pathway under conditions of oxygen-glucose deprivation/reoxygenation. However, in the current study, SNHG12 expression was significantly upregulated after I/R treatment, and was markedly decreased after co-culturing with MSCs. In addition, I/R significantly decreased the proliferation and increased the apoptosis and autophagy of BMECs, and co-culturing with MSCs partially reversed these changes in proliferation, apoptosis, and autophagy induced by I/R. These findings suggest that dysfunctional upregulation of SNHG12 might be positively correlated with the apoptosis and autophagy of BMECs after I/R, and that MSCs alleviate BMEC injuries by targeting SNHG12.

Mesenchymal stem cells have been widely investigated as a treatment for I/R injuries. Tang et al. reported that MSCs could inhibit aquaporin-4 to maintain blood-brain barrier integrity after cerebral ischemia (Tang et al., 2015). Liu et al. (2014) showed that MSCs could rescue endothelial cells injured by I/R via tunneling nanotube like structure-mediated mitochondrial transfer. However, the function of SNHG12 in MSCs has rarely been reported. In the current study, SNHG12 was knocked down in MSCs; after which, those MSCs were co-cultured with BMECs. The results showed that silencing of SNHG12 in MSCs significantly rescued the reduction in proliferation and the increases in apoptosis and autophagy induced by I/R, which was better than the effect exerted by traditional MSCs. Wakabayashi et al. demonstrated that intravenously transplanted human MSCs could reduce infarct volume and induce functional improvement and neuroprotection in a MCAO rat model via inducing insulin-like growth factor 1, vascular endothelial growth factor, and epidermal growth factor (Wakabayashi et al., 2010). Hiroyuki et al. (2011) showed that bone marrow-derived MSCs could reduce apoptosis in hepatic cells to alleviate a hepatic I/R injury. In the current study, MSCs were shown to reduce apoptosis and infarct volume in MACO rat brain tissue, and silencing of SNHG12 in MSCs resulted in a better ameliorative effect than that produced by traditional MSCs. Taken together, our data showed that silencing of SNHG12 in MSCs enhanced the therapeutic effects of those MSCs in treating a cerebral I/R injury.

The PI3K/AKT/mTOR signaling pathway is an important pathway involved in regulating the effects produced by cerebral I/R. Chen et al. revealed that brain-derived neurotrophic factor exerts a neuroprotective effect via the PI3K/AKT/mTOR signaling pathway (Chen et al., 2013). Li et al. (2015) reported that triptolide and 2,4-diamino-6-hydroxypyrimidine exerted neuroprotective effects by inhibiting apoptosis and activating the PI3K/AKT/mTOR signaling pathway. Moreover, a recent study showed that transplantation of bone marrow derived MSCs could alleviate intracerebral hemorrhage by increasing expression of GAP-43 in the PI3K/AKT pathway (Cui et al., 2017). In the current study, we showed that I/R treatment significantly decreased the phosphorylation of PI3K, AKT, and mTOR proteins, and that MSCs significantly inhibited the reductions in PI3K, AKT, and mTOR phosphorylation. In addition, silencing of SNHG12 in MSCs significantly enhanced the effect of those MSCs in activating the PI3K/AKT/mTOR signaling pathway both *in vitro* and *in vivo*. These findings suggest that silencing of SNHG12 in MSCs might enhance the ability of those MSCs to ameliorate I/R injuries via the PI3K/AKT/mTOR signaling pathway.

## Conclusion

In conclusion, I/R treatment significantly increased the expression of SNHG12 in BMECs, and co-culturing with MSCs markedly decreased the levels of SNHG12 expression in BMECs induced by I/R. The results of *in vivo* and *in vitro* experiments showed that silencing of SNHG12 in MSCs significantly enhanced the effectiveness of MSCs in promoting cell proliferation and reducing cell apoptosis and autophagy by activating the PI3K/AKT/mTOR signaling pathway. Thus, downregulation of SNHG12 expression in MSCs might represent a new therapeutic approach for treating for cerebral I/R injuries. However, further investigations are still required to confirm the clinical value of this approach.

## Ethics Statement

Ethics approval and consent to participate: all experimental procedures were approved by the Southern Medical University Ethics Committee and were performed in accordance with the guidelines of the National Institutes of Health on the Care and Use of Animals.

## Author Contributions

YL, SG, and CD conceived and designed the study. YL, SG, WL, TJ, XL, XH, XZ, HS, and NZ analyzed and interpreted the data. YL and CD drafted the manuscript and checked the important intellectual content. All authors critically read and revised the manuscript, and approved the final version of the manuscript for submission.

## Conflict of Interest Statement

The authors declare that the research was conducted in the absence of any commercial or financial relationships that could be construed as a potential conflict of interest.

## Supplementary Material

FIGURE S1 Silencing SNHG12 promote the apoptosis and inhibit the proliferation of BMECs after I/R. **(A)** Relative expression of SNHG12 in BMECs as determined by qRT-PCR. **(B)** Apoptosis of BMECs as determined by flow cytometry. **(C)** Cell proliferation as determined by the EdU assay. The percentage of the M2 peak was analyzed as the percentage of Edu-positive cells. I/R, ischemia/reperfusion; NC, negative control. Comparisons among multiple groups were assessed using ANOVA followed by Turkey’s multiple comparisons test. Compared with the control group, ^∗∗^*P* < 0.01.
