# Supplementary figures and images for: Silencing of SNHG12 Enhanced the Effectiveness of MSCs in Alleviating Ischemia/Reperfusion Injuries via the PI3K/AKT/mTOR Signaling Pathway
Source: Front Neurosci. 2019 Jun 25;13:645. doi: 10.3389/fnins.2019.00645 (PMC6603177; doi:10.3389/fnins.2019.00645)

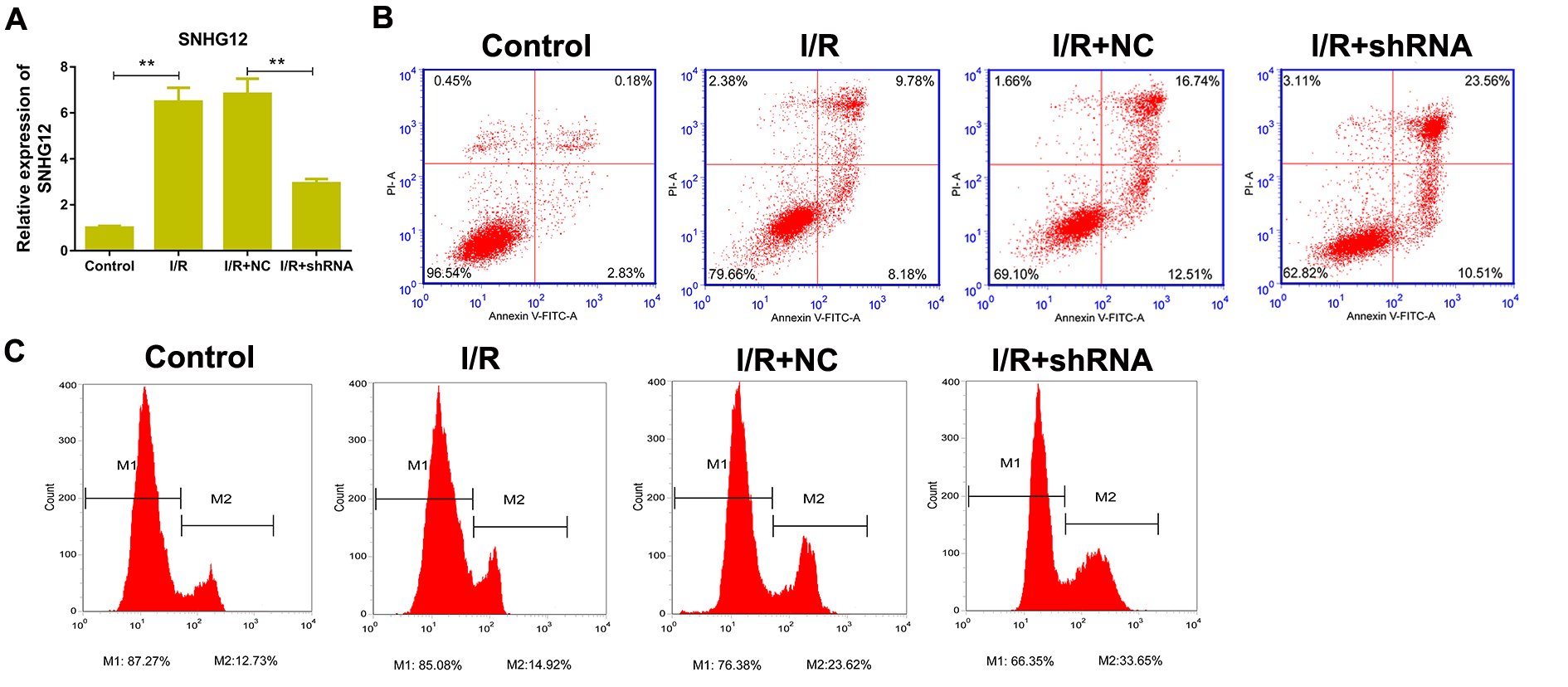

Supplement: FIGURE S1 — Silencing SNHG12 promote the apoptosis and inhibit the proliferation of BMECs after I/R. (A) Relative expression of SNHG12 in BMECs as determined by qRT-PCR. (B) Apoptosis of BMECs as determined by flow cytometry. (C) Cell proliferation as determined by the EdU assay. The percentage of the M2 peak was analyzed as the percentage of Edu-positive cells. I/R, ischemia/reperfusion; NC, negative control. Comparisons among multiple groups were assessed using ANOVA followed by Turkey’s multiple comparisons test. Compared with the control group, ∗∗P < 0.01. [file Image_1.tif]
